# Beyond Glycated Hemoglobin (HbA1c): A Systematic Review of Metabolic Interventions, Insulin Resistance, and Cognitive Function Across the Obesity-to-Diabetes Continuum

**DOI:** 10.7759/cureus.115012

**Published:** 2026-08-23

**Authors:** Eemaz Nathaniel, Abul Kashem Himon, Rana Daniyal Khalid, Ojas Ahuja, Raja Faizan Aurangzeb, Swaira Kanwal, Vishal Gowda, Prasanth Nemani

**Affiliations:** 1 Medicine, University Hospital Wishaw, Wishaw, GBR; 2 Internal Medicine, University Hospital Wishaw, Wishaw, GBR; 3 Internal Medicine, Fauji Foundation Hospital, Islamabad, PAK; 4 Internal Medicine, Quaid-e-Azam Medical College, Bahawalpur, PAK; 5 Internal Medicine, Jackson Memorial Hospital, Miami, USA; 6 Internal Medicine, All Saints University School of Medicine, Roseau, DMA

**Keywords:** cognitive decline, cognitive function, insulin resistance, metabolic interventions, mild cognitive impairment, obesity, systematic review, type 2 diabetes mellitus

## Abstract

Insulin resistance may contribute to cognitive vulnerability before persistent hyperglycemia becomes apparent; however, its clinical relevance as a predictor of cognitive change or therapeutic target remains uncertain. This systematic review with narrative synthesis evaluated the effects of metabolically targeted interventions on insulin resistance and cognitive function in middle-aged and older adults across the obesity-to-type 2 diabetes continuum. MEDLINE via PubMed, Embase, Scopus, and the Cochrane Central Register of Controlled Trials (CENTRAL) were searched for studies published from January 1, 2021, to December 31, 2025. The review followed the Preferred Reporting Items for Systematic Reviews and Meta-Analyses (PRISMA) 2020 guidelines. Eligible designs included randomized controlled trials, prospective randomized parallel-group studies, and longitudinal analyses nested within randomized trials that reported metabolic outcomes alongside cognitive or neuroimaging outcomes. Of the 552 records identified, five studies met the eligibility criteria, comprising four intervention studies and one longitudinal analysis nested within a randomized trial. The interventions comprised lifestyle modification, combined physical and cognitive exercise, dapagliflozin, nutritional supplementation, and moxibustion, with follow-up periods ranging from 12 weeks to two years. Three intervention studies reported concurrent improvements in insulin resistance and cognitive outcomes, whereas one found no significant improvement in insulin resistance and limited neuropsychological benefit. In the single included longitudinal analysis, oral glucose tolerance test (OGTT)-derived measures and the triglyceride-glucose (TyG) index showed more consistent associations with less favorable cognitive or neuroimaging changes than glycated hemoglobin (HbA1c), which demonstrated mixed associations. Three intervention studies raised some concerns regarding risk of bias, one was judged to be at high risk, and the prognostic analysis was assessed as having moderate risk. Overall, measures of insulin resistance may provide information complementary to HbA1c and may have potential as early risk or treatment-response markers for cognitive health. Nevertheless, the small and heterogeneous evidence base, relatively short follow-up periods, and absence of formal mediation analyses preclude causal conclusions. Larger, longer-term, and methodologically rigorous trials are needed to determine whether improving insulin sensitivity can meaningfully alter cognitive trajectories.

## Introduction and background

Cognitive decline is an increasingly important concern among middle-aged and older adults with obesity, metabolic syndrome, and type 2 diabetes mellitus (T2DM). Although chronic hyperglycemia and vascular complications are established contributors to adverse cognitive outcomes, cognitive vulnerability may emerge before overt diabetes or persist despite apparently adequate glycemic control [1]. Insulin resistance has therefore attracted growing attention as a potential link between metabolic dysfunction and cognitive health. Peripheral insulin resistance is associated with compensatory hyperinsulinemia, dyslipidemia, inflammation, endothelial dysfunction, and impaired vascular regulation, each of which may affect brain structure and function. Nevertheless, peripheral insulin resistance should not be assumed to directly reflect insulin resistance in the brain, and its independent contribution to cognitive change remains incompletely understood [2].

Glycated hemoglobin (HbA1c) is central to the diagnosis and monitoring of diabetes because it reflects average glycemic exposure over the preceding two to three months. However, it does not directly characterize insulin sensitivity, compensatory insulin secretion, postprandial glucose regulation, or short-term glycemic variability. Measures such as the homeostatic model assessment of insulin resistance (HOMA-IR), fasting insulin, triglyceride-glucose (TyG) index, and oral glucose tolerance test (OGTT) parameters may capture complementary dimensions of metabolic dysfunction [3]. These abnormalities may influence cognition through altered cerebral glucose utilization, cerebrovascular injury, oxidative stress, chronic inflammation, impaired neurotrophic signaling, and changes in amyloid metabolism. Although these mechanisms provide biological plausibility, their relative contributions in humans remain uncertain, and associations between metabolic markers and cognitive outcomes do not establish causality [4].

Lifestyle, nutritional, pharmacological, and complementary interventions offer an opportunity to determine whether improvements in metabolic health are accompanied by measurable cognitive benefits. Exercise, dietary modification, multidomain lifestyle programs, nutritional supplementation, and glucose-lowering therapies may improve insulin sensitivity while also affecting blood pressure, lipid metabolism, body composition, physical fitness, inflammation, and neurotrophic pathways. Consequently, concurrent metabolic and cognitive improvements may reflect a shared response to broader cardiometabolic changes rather than a direct causal relationship [5]. Interpretation of the clinical literature is further complicated by variations in baseline metabolic and cognitive status, intervention duration, comparator conditions, and cognitive assessment methods. A structured synthesis is therefore needed to distinguish the potential roles of insulin resistance as a risk indicator, treatment-response marker, and mediator of cognitive change [6].

This systematic review evaluated the effects of metabolically targeted interventions on insulin resistance and cognitive function among middle-aged and older adults across the obesity-to-T2DM continuum. It included randomized and prospective controlled intervention studies reporting both metabolic and cognitive outcomes, together with relevant longitudinal analyses nested within randomized trials. Particular attention was given to whether changes in insulin resistance accompanied cognitive changes, whether insulin-related measures provided information complementary to HbA1c, and whether findings varied by intervention type, metabolic stage, cognitive domain, and methodological quality. The review aimed to clarify the clinical significance of insulin resistance as an early risk indicator, treatment-response marker, or potential therapeutic target for cognitive health.

## Review

Materials and methods

Review Design and Reporting

This systematic review evaluated the effects of metabolically targeted interventions on insulin resistance and cognitive function among adults across the obesity-to-T2DM continuum. Before study selection, a protocol was developed specifying the review question, eligibility criteria, outcomes, search strategy, and synthesis methods. The review was conducted and reported in accordance with the Preferred Reporting Items for Systematic Reviews and Meta-Analyses (PRISMA) 2020 statement. Study identification, duplicate removal, screening, eligibility assessment, and final inclusion were documented in a PRISMA 2020 flow diagram [7].

Review Question and PICO Framework

The review addressed the following question: Among middle-aged and older adults with obesity, metabolic syndrome, dysglycemia, prediabetes, insulin resistance, or T2DM, what effects do lifestyle, nutritional, pharmacological, complementary, or multidomain metabolic interventions have on insulin resistance and cognitive function, and are baseline or intervention-related metabolic markers associated with subsequent cognitive change? According to the Population, Intervention, Comparator, and Outcome (PICO) framework, the population comprised metabolically at-risk adults; interventions included strategies intended to improve metabolic health or insulin sensitivity; comparators included placebo, usual care, waiting-list control, health advice, or an active intervention; and outcomes comprised measures of insulin resistance or glucose regulation alongside cognitive, neuropsychological, or neuroimaging outcomes.

Information Sources and Search Period

A comprehensive literature search was conducted in MEDLINE via PubMed, Embase, Scopus, and the Cochrane Central Register of Controlled Trials (CENTRAL). A five-year publication window was applied, covering studies published from January 1, 2021, to December 31, 2025. The reference lists of included studies and relevant reviews were also screened to identify additional eligible records. No geographical or language restrictions were imposed, provided that sufficient information was available from the full text, an English-language abstract, or a reliable translation.

Search Strategy

The search combined controlled vocabulary, including Medical Subject Headings (MeSH) and Emtree terms, with relevant free-text keywords. Terms relating to metabolic dysfunction included “insulin resistance,” “HOMA-IR,” “fasting insulin,” “TyG,” “triglyceride-glucose index,” “OGTT,” “dysglycemia,” “prediabetes,” “HbA1c,” and “type 2 diabetes.” Cognitive terms included “cognition,” “cognitive decline,” “cognitive impairment,” “memory,” “executive function,” “attention,” “processing speed,” “mild cognitive impairment,” “Montreal Cognitive Assessment,” “MoCA,” “Mini-Mental State Examination,” “MMSE,” “neuropsychological,” and “neuroimaging.” Intervention and study-design terms included “intervention,” “randomized controlled trial,” “clinical trial,” “lifestyle,” “exercise,” “diet,” “nutrition,” “pharmacological,” “glucose-lowering treatment,” “supplementation,” “moxibustion,” and “multidomain intervention.” The term “moxibustion” was included as a supplementary free-text term to improve the sensitivity of the search for complementary interventions, which may not be consistently indexed under broader clinical-trial or metabolic-intervention headings. Synonymous terms within each concept were combined using the Boolean operator OR, and the metabolic, cognitive, and intervention concepts were combined using AND. Where appropriate, NOT was used to exclude animal-only and preclinical studies. Subject headings, spelling variants, truncation, and syntax were adapted for each database, and the publication-date limits were applied consistently.

Inclusion Criteria

Studies were eligible if they enrolled middle-aged or older adults with obesity, metabolic syndrome, impaired glucose regulation, prediabetes, insulin resistance, or T2DM, with or without cognitive impairment. Eligible studies evaluated a lifestyle, nutritional, pharmacological, complementary, or multidomain metabolic intervention compared with usual care, placebo, a waiting-list control, health advice, or an active intervention. Studies were required to report at least one measure of insulin resistance or glycemic regulation, such as HOMA-IR, fasting insulin, OGTT-derived measures, TyG index, fasting glucose, or HbA1c, together with at least one cognitive, neuropsychological, or neuroimaging outcome. Eligibility was determined independently of the direction or statistical significance of the findings.

Eligible Study Designs

Randomized controlled trials, randomized open-label trials, and prospective randomized parallel-control studies were eligible. Longitudinal secondary analyses nested within randomized intervention trials were also included when they examined metabolic markers in relation to subsequent cognitive or neuroimaging change.

Exclusion Criteria

Animal and preclinical studies, purely cross-sectional studies, conventional observational cohorts, case reports, case series, reviews, protocols, and conference abstracts without sufficient outcome data were excluded. Studies were also excluded if they focused exclusively on type 1 diabetes, established dementia, acute neurological illness, or conditions unrelated to metabolic dysfunction. Studies that did not report both a relevant metabolic measure and a cognitive or brain-related outcome were not eligible.

Study Selection

All records retrieved from the databases were combined, and duplicates were removed electronically and confirmed manually. Two reviewers independently screened titles and abstracts against the prespecified eligibility criteria. Potentially relevant articles were retrieved in full and independently assessed by the same reviewers. Disagreements at either stage were resolved through discussion and consensus, with consultation of a third reviewer when agreement could not be reached. Reasons for exclusion at the full-text stage were documented and reported in the PRISMA flow diagram [7].

Data Extraction

Data were extracted using a standardized form developed for this review. One reviewer performed the initial extraction, and a second reviewer independently checked all entries against the full-text article. Extracted information included study design, sample size, metabolic and cognitive characteristics of the population, intervention and comparator, intervention duration, follow-up period, insulin-resistance or glycemic measures, cognitive and neuroimaging outcomes, principal findings, and reported associations between metabolic and cognitive changes. When several follow-up points were available, post-intervention and longest-follow-up results were recorded separately. Outcomes were extracted regardless of their direction or statistical significance.

Risk-of-Bias Assessment

Risk of bias was assessed independently by two reviewers, with disagreements resolved by consensus. Randomized intervention effects were evaluated using the Cochrane Risk of Bias 2 tool [8], which considers the randomization process, deviations from intended interventions, missing outcome data, outcome measurement, and selection of the reported result. One of the study was evaluated using the Quality in Prognosis Studies tool [9] because its relevant findings concerned prognostic associations between baseline metabolic markers and later cognitive or neuroimaging changes. Risk-of-bias judgments were incorporated into the interpretation of the findings rather than being used as an automatic basis for study exclusion.

Data Synthesis

A structured narrative synthesis was undertaken because the included studies differed substantially in population characteristics, intervention type, comparator, duration, metabolic measures, and cognitive outcomes. Studies were compared according to whether they demonstrated concordant improvement in metabolic and cognitive outcomes, improvement in only one domain, or no significant improvement in either domain. Intervention findings were further grouped as lifestyle, pharmacological, nutritional, or complementary. A meta-analysis was not considered appropriate because the small number of studies and clinical and methodological heterogeneity would have produced a pooled estimate of uncertain interpretability. Particular attention was given to whether insulin resistance functioned as a baseline risk marker, a treatment-response marker, or a potential mediator of cognitive change.

Results

Study Selection Process

**Figure 1:**
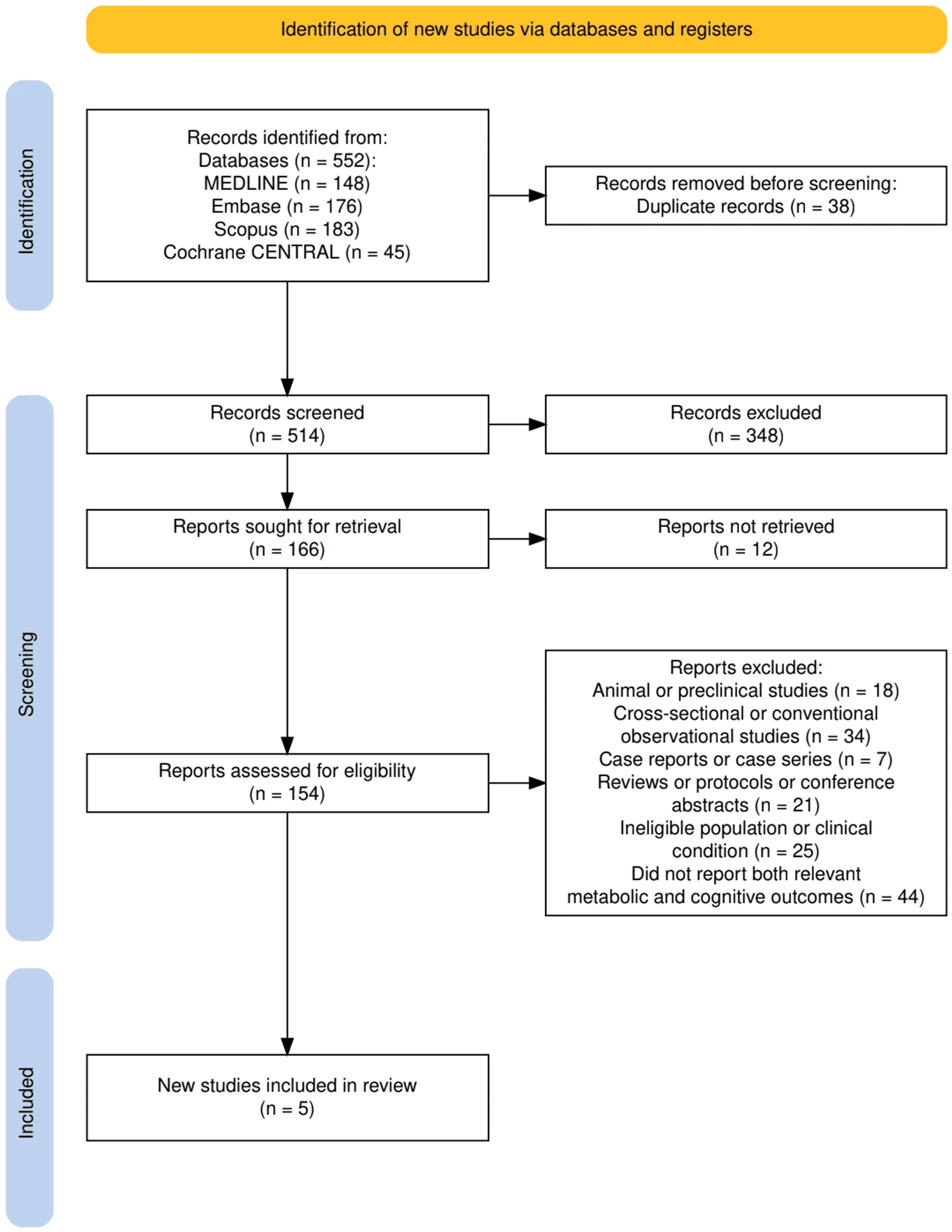
PRISMA flow diagram of the study selection process. PRISMA: Preferred Reporting Items for Systematic Reviews and Meta-Analyses

The study-selection process is presented in Figure 1. Database searching identified 552 records, comprising 148 from MEDLINE, 176 from Embase, 183 from Scopus, and 45 from Cochrane CENTRAL. After removing 38 duplicates, 514 records underwent title and abstract screening, of which 348 were excluded. Full-text retrieval was sought for 166 reports, although 12 could not be obtained, leaving 154 reports for eligibility assessment. Of these, 149 were excluded because they involved animal or preclinical research (n=18), cross-sectional or conventional observational designs (n=34), case reports or case series (n=7), reviews, protocols, or conference abstracts (n=21), ineligible populations or clinical conditions (n=25), or failure to report both relevant metabolic and cognitive outcomes (n=44). Five studies ultimately satisfied the eligibility criteria and were included in the systematic review.

Characteristics of the Selected Studies

The characteristics of the five included studies are summarized in Table 1. Published between 2022 and 2025, these studies had sample sizes ranging from 36 to 1,259 participants and included populations with obesity, an increased risk of dementia, or type 2 diabetes mellitus, with or without cognitive impairment. The interventions comprised dietary modification, combined physical and cognitive exercise, multidomain lifestyle programs, dapagliflozin, branched-chain amino acid supplementation, and moxibustion, with follow-up periods ranging from 12 weeks to two years. Metabolic assessments included HOMA-IR, OGTT-derived measures, the TyG index, fasting glucose, and HbA1c. Cognitive outcomes included global cognition, memory, executive function, processing speed, and neuroimaging measures.

**Table 1:** Characteristics and principal findings of included interventional studies. ACE-III, Addenbrooke’s Cognitive Examination III; BCAA, branched-chain amino acids; FDG-PET, fluorodeoxyglucose positron emission tomography; HbA1c, glycated hemoglobin; HOMA-IR, Homeostatic Model Assessment for Insulin Resistance; MMSE, Mini-Mental State Examination; MoCA, Montreal Cognitive Assessment; MRI, magnetic resonance imaging; OGTT, oral glucose tolerance test; PiB-PET, Pittsburgh compound B positron emission tomography; QIDS, Quick Inventory of Depressive Symptomatology; RCT, randomized controlled trial; ReHo, regional homogeneity; rs-fMRI, resting-state functional magnetic resonance imaging; SAS, Starkstein Apathy Scale; SDMT, Symbol Digit Modalities Test; TMT B-A, Trail Making Test Part B minus Part A; TyG, triglyceride-glucose index.

Study	Design	Intervention and comparator	Duration	Insulin-resistance assessment	Cognitive assessment	Principal metabolic and cognitive findings	Metabolic–cognitive relationship
Keawtep et al. 2024 [10]	Four-arm RCT	Intermittent fasting; physical-cognitive exercise; combined intervention; or no-intervention control	3 months	Fasting insulin and HOMA-IR	MoCA; TMT B-A; Logical Memory; Stroop; verbal fluency; Digit Span	Metabolic: Exercise and combined groups reduced insulin and HOMA-IR versus control. Cognitive: Exercise and combined groups improved memory; only the combined group improved executive function. Diet alone produced no cognitive benefit.	Metabolic and cognitive improvements occurred concurrently, but no formal association or mediation analysis was performed.
Ding et al. 2025 [11]	Randomized prospective parallel-control trial	Dapagliflozin versus control treatment	36 weeks	HOMA-IR, HbA1c, and fasting plasma glucose	MoCA and MMSE; rs-fMRI ReHo in an imaging subgroup	Metabolic: Dapagliflozin reduced HOMA-IR, HbA1c, and fasting glucose. Cognitive: MoCA and MMSE improved; left superior temporal-gyrus ReHo decreased.	MoCA improvement was inversely associated with HOMA-IR (r=−0.653, p=0.006); mediation was not tested.
Lorenzo et al. 2025 [12]	Longitudinal secondary analysis of a multidomain RCT	Multidomain lifestyle intervention versus regular health advice	2 years	OGTT-derived measures; HOMA-IR/HOMA2-IR; TyG; HbA1c	Modified Neuropsychological Test Battery; MRI, PiB-PET, and FDG-PET subgroups	Metabolic: No intervention–control differences occurred in glucose-related markers. Cognitive/neuroimaging: Higher baseline dysglycemia predicted less-favourable cognitive and hippocampal changes; higher TyG predicted greater amyloid accumulation and declining cerebral glucose metabolism.	Baseline markers predicted subsequent outcomes but did not modify the cognitive intervention benefit. No change–change association or mediation was demonstrated.
Matsuda et al. 2022 [13]	Exploratory prospective randomized open blinded-endpoint trial	BCAA, 8 g/day, versus soy protein, 7.5 g/day	24 weeks	HOMA-IR, fasting glucose, fasting insulin, and HbA1c	MMSE; QIDS and SAS for depressive symptoms and apathy	Metabolic: HOMA-IR and glycemic measures did not change or differ between groups. Cognitive: MMSE did not improve. Depressive symptoms improved within the BCAA group, without a significant between-group difference.	Neither insulin resistance nor cognition changed significantly; no metabolic-cognitive association was tested.
Ye et al. 2025 [14]	Randomized wait-list-controlled trial	Huayu Tongluo moxibustion plus routine hypoglycemic treatment versus routine treatment with delayed moxibustion	12 weeks plus 12-week follow-up	HOMA-IR at baseline, post-treatment, and follow-up	MoCA; MMSE; ACE-III; SDMT	Metabolic: HOMA-IR decreased in both groups but was significantly lower with moxibustion after treatment and at follow-up. Cognitive: Moxibustion produced greater improvements in global cognition, attention, memory, verbal fluency, and processing speed than the waiting group.	HOMA-IR and cognition improved concurrently, but no correlation, change-change, or mediation analysis was reported.

Risk of Bias Assessment

The risk-of-bias assessments are presented in Table 2. Four intervention studies were evaluated using the Cochrane Risk of Bias 2 (RoB 2) tool; three were judged to raise some concerns, and one was considered at high risk of bias. The principal concerns included incomplete reporting of allocation concealment, limited blinding, potential practice and attention effects, and post-randomization exclusions. The nested prognostic analysis was evaluated using the Quality In Prognosis Studies (QUIPS) tool and was judged to have a moderate risk of bias, primarily because of potential residual confounding, multiple comparisons, and small neuroimaging subsamples. These assessments were incorporated into the interpretation of the consistency and certainty of the findings.

**Table 2:** Summary risk-of-bias assessment of included studies. RoB 2, Cochrane Risk of Bias 2 tool; QUIPS, Quality in Prognosis Studies tool.

Study	Assessment tool	Overall risk of bias	Key considerations
Keawtep et al. 2024 [10]	Cochrane RoB 2	Some concerns	Blinding was difficult because of the lifestyle intervention, and assessor blinding was unclear. Missing data and outcome reporting appeared acceptable.
Ding et al. 2025 [11]	Cochrane RoB 2	Some concerns	Randomization was reported, but allocation concealment and blinding were insufficiently described. Attrition was reasonably balanced between groups.
Lorenzo et al. 2025 [12]	QUIPS	Moderate risk	Measurements were robust, but the relevant associations were observational and potentially affected by residual confounding, multiple comparisons, and small imaging subsamples.
Matsuda et al. 2022 [13]	Cochrane RoB 2	Some concerns	The trial was open-label, but outcome assessment was blinded and missing data appeared limited. Allocation concealment remained unclear.
Ye et al. 2025 [14]	Cochrane RoB 2	High risk	The open wait-list design, unclear assessor blinding, post-randomization exclusions, and possible attention and practice effects reduce confidence in the findings.

Discussion

Principal Findings

This review identified a limited but clinically relevant body of evidence suggesting that metabolically targeted interventions may produce concurrent improvements in insulin resistance and cognitive function among adults across the obesity-to-diabetes continuum. Improvements in both domains were reported by Keawtep et al. [10], Ding et al. [11], and Ye et al. [14], whereas Matsuda et al. [13] found no significant improvement in insulin resistance and little evidence of neuropsychological benefit. Lorenzo et al. [12] further showed that baseline abnormalities in glucose regulation were associated with less favorable cognitive and neuroimaging trajectories. Collectively, these findings suggest a potentially meaningful association between metabolic and cognitive health. However, the small number and substantial heterogeneity of the included studies preclude firm conclusions regarding causality or overall treatment efficacy.

Paired Metabolic and Cognitive Responses

Evaluating metabolic and cognitive outcomes together offers greater clinical insight than examining either domain in isolation. Parallel improvements in HOMA-IR and cognitive performance across several studies suggest that enhanced insulin sensitivity may accompany cognitive benefit [15]. Conversely, Matsuda et al. [13] found no significant changes in either HOMA-IR or neuropsychological performance, indicating that interventions with plausible metabolic effects do not necessarily produce measurable cognitive benefits. The observed concordant and discordant responses support further investigation of insulin resistance as a potential treatment-response marker. Nevertheless, the current evidence does not establish that the magnitude of metabolic improvement reliably predicts the extent of cognitive improvement [16].

Insulin Resistance Beyond HbA1c

The findings highlight the distinction between chronic glycemic exposure and the broader metabolic disturbances associated with insulin resistance. Although HbA1c is an established measure of average glycemia, it does not fully capture compensatory hyperinsulinemia, postprandial dysregulation, or early reductions in insulin sensitivity. In the analysis by Lorenzo et al. [12], OGTT-derived measures and the TyG index showed more consistent associations with adverse cognitive and neuroimaging changes, whereas HbA1c demonstrated mixed associations. Insulin-related and dynamic glucose measures may therefore provide complementary information when assessing metabolic vulnerability relevant to cognition. However, current evidence is insufficient to demonstrate their superiority to HbA1c or support their routine use in predicting cognitive risk.

Marker, Mediator, or Therapeutic Target?

A central question arising from this review is whether insulin resistance functions as a risk marker, a treatment-response marker, or a causal mediator of cognitive change. The longitudinal findings of Lorenzo et al. [12] support the potential value of metabolic abnormalities as early risk indicators, while the intervention studies suggest that HOMA-IR may reflect metabolic responses to treatment. Evidence supporting a mediating role is substantially weaker. Concurrent improvements in insulin resistance and cognition do not establish that one caused the other, particularly because the interventions may also have affected body composition, lipid levels, blood pressure, physical fitness, inflammation, or neurotrophic signaling. Formal mediation analyses are therefore needed before insulin resistance can be considered a mediator or surrogate endpoint of cognitive benefit [17].

Intervention-Specific Effects

The findings appeared to vary according to the nature of the intervention. In Keawtep et al. [10], exercise and combined interventions improved memory alongside HOMA-IR, whereas dietary modification alone did not produce significant cognitive benefits. This pattern suggests that metabolic improvement may be more relevant to cognition when accompanied by the physiological, neurotrophic, or cognitively stimulating effects of exercise. Ding et al. [11] reported improvements in cognition, HOMA-IR, glycemic control, cholesterol levels, and functional neuroimaging findings following dapagliflozin treatment. However, these concurrent changes prevent the observed cognitive benefits from being attributed specifically to improved insulin sensitivity. Ye et al. [14] also reported favorable metabolic and cognitive outcomes following moxibustion, but the open wait-list design and other methodological limitations reduce confidence in the magnitude and specificity of these effects.

Cognitive Domains and Metabolic Stage

The assessed outcomes varied across studies and included global cognition, memory, executive function, attention, processing speed, and neuroimaging measures. The domain-specific improvements reported by Keawtep et al. [10] suggest that memory and executive function may respond differently to metabolic and exercise-based interventions, whereas Ding et al. [11] and Ye et al. [14] primarily reported changes in global cognitive screening measures. Interpretation is complicated by differences in assessment instruments, baseline cognitive status, practice effects, and potential ceiling effects. The study populations also ranged from adults with obesity but without diabetes to those with established T2DM and cognitive impairment. Metabolic stage and baseline cognitive reserve may therefore influence the detectability and magnitude of cognitive responses; however, the current evidence does not reliably identify which subgroups are most likely to benefit [18].

Strength and Clinical Interpretation of the Evidence

The findings should be interpreted in light of several methodological limitations. Most trials raised concerns regarding risk of bias, and the findings of Ye et al. [14] were considered particularly vulnerable to the absence of blinding, unequal attention between groups, and post-randomization exclusions. Sample sizes were generally modest, follow-up periods were relatively short, and the metabolic and cognitive outcomes were not standardized across studies. Furthermore, the relevant findings from Lorenzo et al. [12] represented observational associations within a randomized controlled trial cohort rather than randomized intervention effects. Although these limitations do not invalidate the observed associations, they substantially restrict causal interpretation. Importantly, short-term improvements in cognitive test performance should not be equated with the prevention of cognitive decline, which requires longer follow-up and evidence of sustained cognitive preservation.

Implications and Future Directions

Future trials should assess HbA1c alongside fasting insulin, HOMA-IR, the TyG index, and OGTT-derived measures to determine which markers most reliably identify cognitive vulnerability and treatment response. Cognitive assessments should incorporate standardized domain-specific batteries, blinded assessors, alternate test forms, and follow-up periods sufficient to distinguish transient improvements in test performance from sustained cognitive preservation [19]. Studies should also evaluate vascular, inflammatory, neurotrophic, and neuroimaging pathways and apply formal mediation analyses to determine whether changes in insulin resistance account for observed cognitive effects. Current evidence suggests that insulin resistance may complement HbA1c in research-based assessments of metabolically related cognitive risk. However, its role as a validated predictor, causal mediator, or therapeutic target for cognitive outcomes remains to be established.

## Conclusions

Current evidence suggests that insulin resistance may be associated with cognitive outcomes across the obesity-to-type 2 diabetes continuum. Several interventions that improved insulin sensitivity also produced cognitive benefits, particularly those incorporating exercise or achieving broader cardiometabolic improvements. However, these concurrent changes do not establish insulin resistance as the mechanism underlying cognitive improvement. HbA1c alone may not fully capture metabolically related cognitive vulnerability, while HOMA-IR, the TyG index, and OGTT-derived measures may provide complementary information. Overall, insulin resistance shows promise as an early risk indicator and treatment-response marker but has not been validated as a surrogate endpoint for cognitive outcomes. Larger, longer-term trials incorporating standardized cognitive assessments and formal mediation analyses are needed to determine whether targeting insulin resistance can meaningfully alter cognitive trajectories.
